# Hepatic Alveolar Echinococcosis Mimicking Hepatic Malignancy

**DOI:** 10.1590/0037-8682-0346-2024

**Published:** 2026-02-09

**Authors:** Sadullah Şimşek, Mehmet Salih Karaca, Tarık Sağlam

**Affiliations:** 1 Gazi Yasargil Training and Research Hospital, Department of Radiology, Diyarbakır, Türkiye.

**FIGURE 1:**
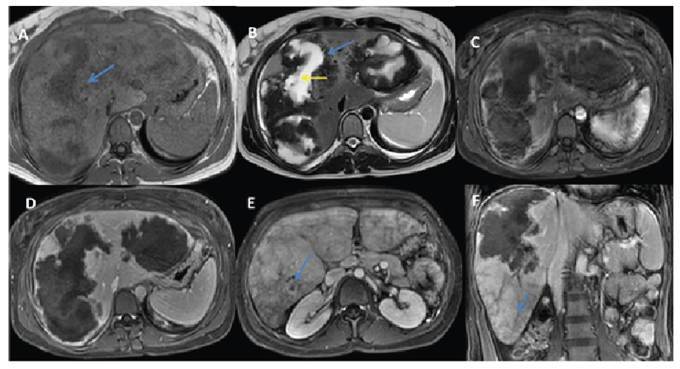
Magnetic resonance imaging findings. **(A)** T1-weighted images show hypointense lesions **(blue arrow)**, while **(B)** T2-weighted images demonstrate a hypointense peripheral rim **(blue arrow)** with central hyperintensity **(yellow arrow)**. Mild peripheral contrast enhancement is visible in the arterial phase **(C)**, becoming more pronounced in the delayed phases **(D)**. Perfusion reduction secondary to portal vein thrombosis **(E, blue arrow)**, along with a heterogeneous parenchymal appearance **(F, blue arrow)**.

**FIGURE 2:**
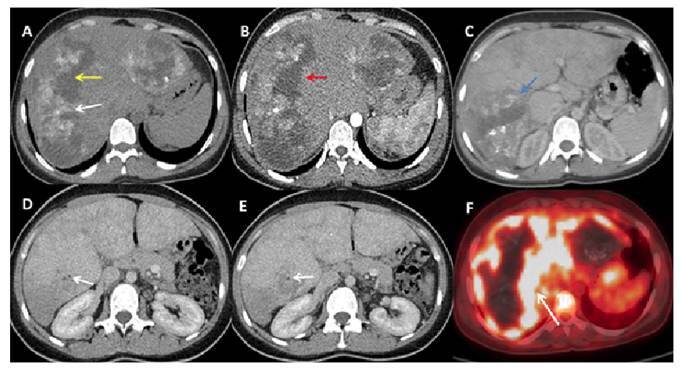
Noncontrast abdominal computed tomography (CT) demonstrates **(A)** hepatic lesions with peripheral calcifications **(white arrow)** and central cystic areas **(yellow arrow)**. Peripheral contrast enhancement is observed during the arterial **(B, red arrow)** and portal venous phases **(C, blue arrow)**. Perfusion reduction secondary to portal vein thrombosis is noted **(D, E, white arrow)**. Positron emission tomography CT shows **(F)** intense fluorodeoxyglucose uptake around the lesions **(white arrow).**

A 40-year-old woman was referred for evaluation of hepatic masses detected on ultrasonography. Magnetic resonance imaging demonstrated two lesions that were hypointense on T1-weighted images and exhibited a hypointense rim with central hyperintensity on T2-weighted images. Persistent peripheral contrast enhancement was observed across all dynamic phases, accompanied by perfusion abnormalities in the surrounding hepatic parenchyma (Figure 1). Computed tomography (CT) confirmed the presence of peripheral calcifications, invasion of the posterior branch of the right portal vein, and markedly reduced perfusion (Figure 2). Positron emission tomography CT revealed intense fluorodeoxyglucose uptake around the lesions (SUVmax: 8.9), consistent with active inflammation. Collectively, these findings suggested hepatic alveolar echinococcosis (HAE), which was subsequently confirmed by positive serology.

HAE, caused by *Echinococcus multilocularis*, is a rare but potentially life-threatening zoonotic disease. Its infiltrative growth pattern often mimics malignant neoplasms^1^. Radical resection combined with antiparasitic therapy may be curative, whereas long-term albendazole treatment can help control disease progression in cases where surgery is not feasible^2^.
